# Acute Encephalopathy in Multiple Sclerosis With Renal Failure: Synergistic Neurotoxicity of Baclofen and Meropenem

**DOI:** 10.1155/crdi/7420497

**Published:** 2026-06-19

**Authors:** Bernacchia D., Rigoni M., Canavesi G., Franzetti M., Pagani G., Bassani F., Verrengia E., Gottardo R., Di Toma L. F., Pavia C., Rusconi S.

**Affiliations:** ^1^ Infectious Diseases Unit, ASST-Ovest Milanese, Legnano, Milan, Italy; ^2^ Department of Biomedical and Clinical Sciences, DIBIC, University of Milan, Milan, Italy, unimi.it; ^3^ Neurology Unit, ASST-Ovest Milanese, Legnano, Milan, Italy; ^4^ Department of Diagnostics and Public Health, Unit of Forensic Medicine, University of Verona, Verona, Italy, univr.it; ^5^ Nephrology Unit, ASST-Ovest Milanese, Legnano, Milan, Italy; ^6^ Microbiology Unit, ASST-Ovest Milanese, Legnano, Milan, Italy

## Abstract

The clinical case of a 62‐year‐old male with a history of secondary progressive multiple sclerosis (MS), who developed acute encephalopathy during antibiotic therapy with meropenem for a urinary tract infection (UTI) by Proteus mirabilis ESBL‐producing is reported here. The clinical course revealed an altered level of consciousness, seizures, and electroencephalographic abnormalities, which were initially attributed to antibiotic toxicity. However, further investigation revealed an overdose of baclofen, a muscle relaxant the patient had been taking for several years, leading to the diagnosis of acute metabolic encephalopathy. Moreover, given the patient’s impaired renal function, a possible interaction between baclofen and meropenem neurotoxicity was suspected, likely related to their combined effects on GABA receptors. While baclofen and meropenem neurotoxicity are well‐documented individually, their potential interaction remains poorly understood, particularly in patients with altered renal function. This case highlights the importance of considering drug toxicity and interactions in patients with altered mental status, especially in those with polypharmacy and comorbid conditions.

## 1. Introduction

Metabolic encephalopathy is a multifactorial condition that represents a significant cause of altered mental status characterized by a spectrum of neurological symptoms ranging from mild cognitive impairment to deep coma, resulting from a variety of metabolic disturbances, infections, and the use of specific drugs. While the pathophysiology is complex, it primarily involves alterations in cerebral metabolism due to systemic factors such as electrolyte imbalances, liver or renal dysfunction, and infections. Of particular concern is the role of pharmacological agents, including antibiotics, which are implicated in both direct and indirect mechanisms of neuronal dysfunction [1].

Multiple sclerosis (MS) is a chronic autoimmune neurological disease of central nervous system (CNS) characterized by demyelinating plaques involving brain and spinal cord. MS symptoms often include fatigue, vision problems, muscle weakness, ataxia, dizziness, bladder dysfunction, and rarely seizures. MS brain lesions have a potential role in triggering electrical instability [2]. Spinal cord involvement often causes neurological bladder failure with chronic urinary retention, incontinence, and an increased risk of recurrent urinary tract infections (UTIs) [3, 4]. As a result, these individuals are disproportionately exposed to antimicrobial therapies, hospitalizations, and infections caused by multidrug‐resistant (MDR) organisms. The repeated need for hospitalization due to these infections significantly burdens healthcare systems and contributes to the widespread use of broad‐spectrum antibiotics, particularly carbapenem. While carbapenem remains a cornerstone of therapy for MDR infections, their extensive use raises concerns regarding antimicrobial resistance and potential adverse effects, including neurotoxicity. These neurotoxic effects include seizures and encephalopathy [5] and are mainly observed with drugs like imipenem–cilastatin or ertapenem but also with meropenem, one of the most prescribed carbapenem [6]. The mechanism of carbapenem‐induced neurotoxicity is thought to involve the inhibition of gamma‐aminobutyric acid (GABA)‐A receptors and the disruption of synaptic transmission, leading to excitatory neurotoxicity [7, 8]. Such neurotoxic effects are often exacerbated in patients with renal impairment, where drug clearance is reduced, increasing the risk of toxicity [9]. In clinical practice, it is not common to determine the CNS concentration and pharmacokinetic of meropenem because it needs a diagnostic lumbar puncture and because the intracerebral therapeutic levels of carbapenem are not the same as in cerebrospinal fluid (CSF).

Similarly, baclofen, a GABA‐B agonist, is widely used in the management of spasticity, particularly in conditions like MS [10]. However, it carries a well‐documented risk of CNS toxicity, including encephalopathy, particularly when overdosed or administered in patients with impaired renal function [11, 12]. In MS patients, who often experience complex neurological impairments, the risk of baclofen‐induced encephalopathy is heightened due to altered pharmacokinetics and potentially compromised renal function. Both meropenem and baclofen have a renal clearance: Damage in renal function could lead to drug competition in excretion and possible upstream accumulation [13–16]. Actually, no major pharmacological interactions are described in different online sources [17–19].

This case report describes an event of metabolic encephalopathy induced by baclofen with the possible pharmacological triggering by meropenem in patients with chronic neurological conditions. Little is known about the potential interaction between baclofen and meropenem in contributing to neurotoxicity, especially in patients with impaired renal function. This report describes a case of acute encephalopathy in a 62‐year‐old male, which was initially misattributed to antibiotic therapy but later identified as a consequence of baclofen overdose, with a possible exacerbation due to meropenem coadministration.

## 2. Case Report

### 2.1. Patient Information

The patient, a 62‐year‐old male, was admitted to the Infectious Diseases Unit of Legnano hospital (ASST Ovest Milanese, Milan, Italy) in January 2025 for treatment of a UTI caused by Proteus mirabilis producing extended‐spectrum beta lactamase (ESBL‐producing). His remote medical history is featured by MS diagnosed in 1999 conditioning paraplegia until the end of self‐sufficiency. He had discontinued any disease‐modifying therapy for MS in 2015. His last brain MRI in December 2024 showed no significant changes compared to the previous one in May 2023, indicating nonactive MS, with predominant spinal involvement. The patient showed high motor disability, preserved cognitive functions, weight of 75 kg (BMI 24) with discrete muscle trophism and, due to urinary dysfunction, since May 2023, he carried a suprapubic catheter. Three months prior to this hospitalization, he underwent a left nephrectomy for xanthogranulomatous glomerulonephritis, with no impaired renal function.

### 2.2. Clinical Course

Upon admission, the patient started intravenous meropenem therapy (500 mg every 12 h) due to the UTI. His renal function was initially impaired (serum creatinine 2.52 mg/dL and GFR 26 mL/min), and the antibiotic dose was adjusted accordingly. On the ninth day of antibiotic therapy, the patient’s renal function improved (serum creatinine 1.61 mg/dL and GFR 44 mL/min), and the meropenem dose was increased to 1 g every 12 h. Three hours after receiving the first increased dose, the patient exhibited altered mental status, with drowsiness and lack of response to tactile and verbal stimuli (GCS 7). He has no previous headache, neck stiffness, photophobia, nausea, vomiting, seizures, and head injury. Vital signs remained stable, and he remained afebrile with active diuresis. Arterial blood gas showed mild mixed acidosis (Lactate 1.6), and laboratory tests revealed mild liver enzyme elevation but no signs of inflammation. A neurological consult led to the performance of a baseline brain CT scan, which showed no significant abnormalities, and EEG findings indicated diffuse slow activity without epileptogenic foci.

The patient’s mental status improved spontaneously in the next couple of hours, but he continued to have difficulty with speech. However, after 10 h from the first meropenem 1 g dose, he experienced two episodes of respiratory arrest associated with generalized spastic seizures in the upper limbs and facial muscles. These episodes were promptly managed by the medical and resuscitation team without resulting in hypoxic brain injury. Anticonvulsant therapy with diazepam and levetiracetam was initiated intravenously, and the seizures were controlled without further complications.

### 2.3. Diagnostic Workup

At the moment of the last major neurological event, a diagnostic lumbar puncture was performed, revealing normal cell count and glucose levels (WC 6/μL, RC 340/μL, and glucose 62 mg/dL), the film array for bacterial and viral CNS pathogens shown no significant microbiological isolation (only Staphylococcus capitis grown on CSF at 26 h and 11 min, and it was considered a contaminant). Two blood culture sets were performed (both aerobic and anaerobic were later negative). The patient remained in a somnolent state but stable in terms of vital parameters. An EEG performed 12 h later revealed a burst‐suppression pattern, prompting the need for an urgent brain MRI, which showed no significant pathological findings that could explain the abnormal electrophysiological findings. During the MRI, the patient showed a transitory episode of significant bradycardia with a nadir cardiac rate of 36 bpm, spontaneously solved without atropine.

### 2.4. Multidisciplinary Discussion

Given the absence of neurological abnormalities on MRI imaging and the evolving clinical picture, the case was reviewed by a multidisciplinary team. The initial suspicion was of acute metabolic encephalopathy. A review of the patient’s medication regimen revealed that he had been taking baclofen 25 mg three times daily for spasticity, a dose that had been stable for the last months despite the impaired renal function and the recent nephrectomy. No other drug was suspected of intoxication. Considering his renal function impairment and the recent changes in his clinical condition, baclofen neurotoxicity was suspected. Additionally, given the known neurotoxic potential of meropenem and its ability to interact with GABAergic pathways, a possible interaction between baclofen and meropenem leading to exacerbated neurotoxicity was considered. The Poison Control Center (ICS Maugeri, Pavia) confirmed the likelihood of baclofen toxicity, but the role of meropenem in worsening the neurological symptoms could not be excluded. Serologic and liquor sample were sent to the laboratory of the Unit of Forensic Medicine of the University of Verona to confirm the high levels of drug of baclofen: in CSF after 10 h from the last administration, the level was 472 ng/mL; in serum after 10 h from the last administration, the level was 1548 ng/mL, then 1176 ng/mL after 24 h, and 312 ng/mL after 96 h. Although it was not possible to analyze meropenem in CSF, meropenem serum concentration was already undetectable after 10 h.

### 2.5. Management and Outcome

Meropenem and baclofen were discontinued (the patient conducted enough days of antibiotic therapy for UTI), and the patient received supportive care, including intravenous fluids, diuresis stimulation, and close monitoring. Dialysis was not deemed necessary balancing risk and benefits of the procedure. Over the following hours, the patient showed gradual improvement in his neurological status, and the altered level of consciousness resolved. The patient remained stable, and his condition continued to improve without further seizures or respiratory events.

## 3. Discussion

This case highlights the potential for baclofen overdose in fragile patients with meropenem coadministration, particularly when renal function is compromised.

Using high doses of baclofen does not alarm neurologists during the management of patients with MS or other chronic spasticity syndromes. It is well known that the normal dose is a maximum of 80 mg/day divided into three administrations, but sometimes in clinical practice, it reaches 120 mg/day [20]. Indeed, during chronic consumption, it is often necessary to administer baclofen above the recommended dosages to control spastic symptoms: high dosages of baclofen are usually well tolerated and do not lead to any clinical complication, but several overcoming conditions during acute illnesses had to be considered such as the worsening chronic renal failure and codrug administration. Baclofen is mainly cleared by kidneys, and in case of renal impairment, it needs a drug adjustment to prevent toxicity [11, 21].

The symptoms of baclofen overdose can mimic other neurological conditions, such as encephalopathy or seizure disorders, making diagnosis challenging [22]. For sure, baclofen overdose must be suspected when the daily dose is above 200 mg/day and toxic drug serum concentrations are above 800 ng/mL [23, 24]. In the absence of organic and vascular ipossic damage, EEG findings such as the burst suppression pattern, the generalized suppression or the triphasic pattern are typical of baclofen overdose [25–27].

Furthermore, while carbapenem neurotoxicity is relatively rare and well‐documented, its potential to interact with baclofen and exacerbate GABAergic dysfunction in patients with renal impairment remains underexplored. Both drugs can interfere with the balance of excitatory and inhibitory neurotransmission in the brain. Baclofen’s action on GABA‐B receptors and meropenem’s inhibitory effects on GABA‐A receptors may synergistically impair brain function, leading to more pronounced cognitive and motor disturbances. Therefore, understanding the pharmacodynamic interactions between these drugs is crucial for managing patients with neurological disorders who require both medications. In literature, we found only two different case reports describing baclofen overdose symptoms in patients with chronic spasticity during an ongoing antibiotic therapy with carbapenem: In both documents, there are strong analogies in terms of clinical setting, symptomatologic manifestations, clinical findings, and solutions but neither of them proposes as the main explanation the potential interactions between the two drugs [26, 28]. The data from the reported clinical cases are shown and compared in Table 1.

**TABLE 1:** The main characteristics of three clinical cases with the copresence of neurological disease, renal failure and suspected interaction between baclofen and carbapenem.

Case	Age	Sex	Neurological background	Neurological index event	Renal function	Urinary disfunction	Site of infection	Baclofen dosage	Meropenem dosage	EEG	Toxic baclofen levels	Index event treatment	Intensive care support	Recovery
Justin Gold et al.	35	Male	Cervical spine trauma at 17 y.o, paraplegic in the lower extremities	No spontaneous opening eyes, no following commands, only localizing painful stimuli on upper extremities	Referred acute kidney injury—No data	No data	*E. coli* UTI + WNV exposure	Intrathecal pump 460mcg daily + oral 30 mg q6 h	No data	Burst suppression pattern	No data	Reduction of intrathecal and oral baclofen dosage	Intubation	Yes—improvement Day 3 after intrathecal pump removal
Klimko et al.	55	Female	Multiple Sclerosis since 23 y.o.	Lethargic and less responsive, trouble swallowing, stopped talking, unable to follow commands, jaw rigidity, hyperextended neck	e‐GFR 55 mL/min and chronic hyponatremia (129 mEq/L)	Bladder dysfunction with daily self‐catheterization	ESBL ‐producing *E. coli* UTI‐	40–60 mg daily	1 g q8 h	Negative for seizures	No data	Switch antibiotic therapy and reduction oral baclofen dosage	Nasogastric tube	Yes—improvement at day 3 after 2 days of baclofen suspension and carbapenem suspension
Bernacchia et al.	62	Male	Multiple Sclerosis since 36 y.o.	Initial drowsiness and lack of response to tactile and verbal stimuli ‐> respiratory arrest associated with generalized spastic seizures	Single kidney, eGFR 26 mL/min	Bladder disfunction with suprapubic catheter	ESBL‐ producing *P. mirabilis*	25 mg q8h	1 gr q12 h	Burst suppression pattern	CSF level 472 ng/mL (after 10 h); Serum levels 1.548 ng/mL (after 10 h), 1176 ng/mL (after 24 h) and 312 ng/mL (after 96 h)	Transitory suspension of oral baclofen; meropenem suspension, intravenous fluids, diuresis stimulation	Close monitoring and resuscitation team intervention	Yes—improvement few hours after baclofen suspension

Anyway, it should be useful to consider and evaluate therapeutic levels of both baclofen and meropenem in serum and CSF in critical patients. Meropenem serum/CSF levels are not well representative of the intracerebral therapeutic concentrations, and CNS concentrations are not routinely available because of the necessity of invasive procedures [29].

## 4. Conclusion

Meropenem therapy should be considered as a potential trigger of baclofen toxicity in patients receiving chronic baclofen therapy, particularly in the elderly and those with renal impairment. Due to the life‐threatening condition, the early recognition and discontinuation of the potentially toxic agents, along with supportive care, are essential for a positive outcome. Despite the long experience with meropenem and baclofen, little is known about GABAergic molecular interaction between them, and further studies are needed.

## Funding

Open access publishing facilitated by Aziende Socio Sanitarie Territoriale Ovest Milanese, as part of the Wiley – SBBL agreement.

## Conflicts of Interest

The authors declare no conflicts of interest.

## Data Availability

The data that support the findings of this study are available on request from the corresponding author. The data are not publicly available due to privacy or ethical restrictions.

## References

[1] le Guennec L. , Marois C. , Demeret S. , Wijdicks E. , and Weiss N. , Toxic-Metabolic Encephalopathy in Adults: Critical Discussion and Pragmatical Diagnostic Approach, Revue Neurologique. (2022) 178, no. 1–2, 93–104, 10.1016/j.neurol.2021.11.007. 34996631

[2] Marcus R. , What Is Multiple Sclerosis?, JAMA. (2022) 328, no. 20, 10.1001/jama.2022.14236. 36413229

[3] Medeiros Junior W. L. G. D. , Demore C. C. , Mazaro L. P. , de Souza M. F. N. , Parolin L. F. , Melo L. H. , Junior C. R. W. , and Gonçalves M. V. M. , Urinary Tract Infection in Patients with Multiple Sclerosis: an Overview, Multiple Sclerosis and Related Disorders. (2020) 46, 10.1016/j.msard.2020.102462. 32890816

[4] Campetella M. , Filomena G. , Marino F. , Fantasia F. , Russo P. , Gavi F. , Rossi F. , Gandi C. , Ragonese M. , Foschi N. , Totaro A. , Sacco E. , Racioppi M. , and Bientinesi R. , Etiology, Presentation and Management of Urinary Tract Infections in Multiple Sclerosis Patients: a Review of the Current Literature, Urologia Journal. (2024) 91, no. 2, 384–393, 10.1177/03915603231224511. 38279809

[5] Deshayes S. , Coquerel A. , and Verdon R. , Neurological Adverse Effects Attributable to β-Lactam Antibiotics: a Literature Review, Drug Safety. (2017) 40, no. 12, 1171–1198, 10.1007/s40264-017-0578-2. 28755095

[6] Fernández-Rubio B. , Luque-Márquez R. , and Gil-Navarro M. V. , Probable ertapenem-induced Encephalopathy; Case Report and Suggested Alternatives for Chronic Prostatitis, Daru Journal of Pharmaceutical Sciences. (2022) 30, no. 1, 159–163, 10.1007/s40199-021-00425-5. 35023080 PMC9114266

[7] Hikida M. , Masukawa Y. , Nishiki K. , and Inomata N. , Low Neurotoxicity of LJC 10,627, a Novel 1 Beta-Methyl Carbapenem Antibiotic: Inhibition of gamma-aminobutyric acidA, Benzodiazepine, and Glycine Receptor Binding in Relation to Lack of Central Nervous System Toxicity in Rats, Antimicrobial Agents and Chemotherapy. (1993) 37, no. 2, 199–202, 10.1128/AAC.37.2.199. 8383938 PMC187638

[8] Sunagawa M. , Matsumura H. , Sumita Y. , and Nouda H. , Structural Features Resulting in Convulsive Activity of Carbapenem Compounds: Effect of C-2 Side Chain, Journal of Antibiotics. (1995) 48, no. 5, 408–416, 10.7164/antibiotics.48.408. 7797443

[9] Schliamser S. E. , Cars O. , and Norrby S. R. , Neurotoxicity of beta-lactam Antibiotics: Predisposing Factors and Pathogenesis, Journal of Antimicrobial Chemotherapy. (1991) 27, no. 4, 405–425, 10.1093/jac/27.4.405. 1856121

[10] Ghanavatian S. and Baclofen D. A. , Baclofen, StatPearls. (2024) . 30252293

[11] Wolf E. , Kothari N. R. , Roberts J. K. , and Sparks M. A. , Baclofen Toxicity in Kidney Disease, American Journal of Kidney Diseases. (2018) 71, no. 2, 275–280, 10.1053/j.ajkd.2017.07.005. 28899601

[12] Muanda F. T. , Weir M. A. , Bathini L. , Blake P. G. , Chauvin K. , Dixon S. N. , McArthur E. , Sontrop J. M. , Moist L. , and Garg A. X. , Association of Baclofen with Encephalopathy in Patients with Chronic Kidney Disease, JAMA, the Journal of the American Medical Association. (2019) 322, no. 20, 10.1001/jama.2019.17725. PMC6865230 31705755

[13] Aisen M. L. , Dietz M. A. , Rossi P. , Cedarbaum J. M. , and Kutt H. , Clinical and Pharmacokinetic Aspects of High Dose Oral Baclofen Therapy, The Journal of the American Paraplegia Society. (1992) 15, no. 4, 211–216, 10.1080/01952307.1992.11761520. 1431867

[14] Vlavonou R. , Perreault M. M. , Barrière O. , Shink E. , Tremblay P. O. , Larouche R. , Pichette V. , and Tanguay M. , Pharmacokinetic Characterization of Baclofen in Patients with Chronic Kidney Disease: Dose Adjustment Recommendations, Journal of Clinical Pharmacology. (2014) 54, no. 5, 584–592, 10.1002/jcph.247. 24414993

[15] Thalhammer F. and Hörl W. H. , Pharmacokinetics of Meropenem in Patients with Renal Failure and Patients Receiving Renal Replacement Therapy, Clinical Pharmacokinetics. (2000) 39, no. 4, 10.2165/00003088-200039040-00003. 11069213

[16] van Ginneken C. A. M. and Russel F. G. M. , Saturable Pharmacokinetics in the Renal Excretion of Drugs, Clinical Pharmacokinetics. (1989) 16, no. 1, 38–54, 10.2165/00003088-198916010-00003. 2650954

[17] https://www.drugs.com/drug_interactions.html.

[18] https://reference.medscape.com/drug-interactionchecker.

[19] https://go.drugbank.com/drug-interaction-checker.

[20] Baclofen, 2021, https://www.drugs.com/monograph/baclofen.html.

[21] Wuis E. W. , Dirks M. J. M. , Termond E. F. S. , Vree T. B. , and van der Kleijn E. , Plasma and Urinary Excretion Kinetics of Oral Baclofen in Healthy Subjects, European Journal of Clinical Pharmacology. (1989) 37, no. 2, 181–184, 10.1007/BF00558228. 2792173

[22] Miller J. J. , Baclofen Overdose Mimicking Anoxic Encephalopathy: a Case Report and Review of the Literature, Therapeutic Advances in Drug Safety. (2017) 8, no. 5, 165–167, 10.1177/2042098617693571. 28588762 PMC5444597

[23] Leung N. Y. , Whyte I. M. , and Isbister G. K. , Baclofen Overdose: Defining the Spectrum of Toxicity, Emergency Medicine Australasia. (2006) 18, no. 1, 77–82, 10.1111/j.1742-6723.2006.00805.x. 16454779

[24] Wuis E. W. , Dirks M. J. M. , Vree T. B. , and van der Kleijn E. , Pharmacokinetics of Baclofen in Spastic Patients Receiving Multiple Oral Doses, Pharmaceutisch Weekblad-Scientific Edition. (1990) 12, no. 2, 71–74, 10.1007/BF01970149. 2336342

[25] Farhat S. , el Halabi T. , Makki A. , Atweh S. F. , Nasreddine W. , and Beydoun A. , Coma with Absent Brainstem Reflexes and a Burst Suppression on EEG Secondary to Baclofen Toxicity, Frontiers in Neurology. (2020) 11, 10.3389/fneur.2020.00404. PMC7237569 32477255

[26] Gold J. , Zhao K. , Abraham M. , Behmer Hansen R. , Lad M. , and Mammis A. , Encephalopathy of Unknown Origin in a Baclofen Patient: Case Report and Review of the Literature, World Neurosurgery. (2020) 136, 136–139, 10.1016/j.wneu.2020.01.044. 31954899

[27] Triplett J. , Lawn N. , and Dunne J. , Baclofen Neurotoxicity: a Metabolic Encephalopathy Susceptible to Exacerbation by Benzodiazepine Therapy (P6.356), Neurology. (2016) 86, no. 16_supplement, 10.1212/wnl.86.16_supplement.p6.356. 30688773

[28] Klimko C. v. , Sanders J. M. , and Johns M. L. , Probable Encephalopathy and Spasticity in a Multiple Sclerosis Patient Following Carbapenem Administration: a Case Report and Brief Literature Review, Journal of Pharmacy Practice. (2023) 36, no. 3, 699–704, 10.1177/08971900211063277. 34958618

[29] Alshaer M. H. , Barlow B. , Maranchick N. et al., Meropenem Population Pharmacokinetics and Simulations in Plasma, Cerebrospinal Fluid, and Brain Tissue, Antimicrobial Agents and Chemotherapy. (2022) 66, no. 8, 10.1128/aac.00438-22. PMC9380529 35862739

